# Cytokines and HCV-Related Disorders

**DOI:** 10.1155/2012/468107

**Published:** 2012-05-07

**Authors:** Poupak Fallahi, Clodoveo Ferri, Silvia Martina Ferrari, Alda Corrado, Domenico Sansonno, Alessandro Antonelli

**Affiliations:** ^1^Department of Internal Medicine, School of Medicine, University of Pisa, Via Roma, 67, 56100 Pisa, Italy; ^2^Department of Internal Medicine, Rheumatology Unit, School of Medicine, University of Modena and Reggio Emilia, Via del Pozzo, 71, 41100 Modena, Italy; ^3^Department of Internal Medicine and Clinical Oncology, University of Bari, Piazza Giulio Cesare, 11, 70124 Bari, Italy

## Abstract

Cytokines are intercellular mediators involved in viral control and liver damage being induced by infection with hepatitis C virus (HCV). The complex cytokine network operating during initial infection allows a coordinated, effective development of both innate and adaptive immune responses. However, HCV interferes with cytokines at various levels and escapes immune response by inducing a T-helper (Th)2/T cytotoxic 2 cytokine profile. Inability to control infection leads to the recruitment of inflammatory infiltrates into the liver parenchyma by interferon (IFN)-gamma-inducible CXC chemokine ligand (CXCL)-9, -10, and -11 chemokines, which results in sustained liver damage and eventually in liver cirrhosis. The most important systemic HCV-related extrahepatic diseases—mixed cryoglobulinemia, lymphoproliferative disorders, thyroid autoimmune disorders, and type 2 diabetes—are associated with a complex dysregulation of the cytokine/chemokine network, involving proinflammatory and Th1 chemokines. The therapeutical administration of cytokines such as IFN-alpha may result in viral clearance during persistent infection and reverts this process.

## 1. Introduction

Cytokines are small soluble proteins secreted by immune system cells and other cells and are part of an intercellular communication system responsible for immune response [1]. These proteins play their role by binding specific cell receptors that either induce or inhibit cytokine-regulated genes. During viral infection, various cytokines play a role both in viral clearance and tissue damage [1].

## 2. Cytokines

Over 100 different cytokines have been reported, which are classified according to their primary role. In relation to their functions, cytokines can be classified in subgroups: (a) pro-inflammatory cytokines (interleukin (IL)-1, IL-6, tumor necrosis factor (TNF)-alpha); (b) T-helper (Th)1 cytokines, which are produced by Th1-activated lymphocytes (interferon (IFN)-gamma, IL-12, IL-18); (c) Th2-type cytokine which plays a role in the inhibition of cytokines derived from Th1 cell which turns out to downregulate the function of Th1 immune responses, inhibiting antigen-presenting capacity of macrophage and promoting B-cell proliferation and therefore antibody production (IL-10, IL-4, IL-5, IL-13); (d) Th17 cytokines which are important for the differentiation of Th17 lymphocytes. IL-23, together with IL-6 and transforming growth factor (TGF)-beta, leads to the differentiation of Th0 to Th17 cells which carry out the function of secreting IL-17A, IL-17F, TNF-alpha, and IL-1 thus leading to proinflammatory reaction [1].

## 3. Chemokines

Chemokines are a large multifunctional family of cytokines (chemotactic cytokines) that induce the migration of cells to sites of infection or injury. Functionally chemokines fall into two main categories: homeostatic or proinflammatory. Homeostatic chemokines are produced constitutively; these are generally involved in lymphocyte trafficking, immune surveillance, and localization of lymphocytes with antigen in the lymphatic system [2]. Other chemokines are only produced by cells during infection or following a proinflammatory stimulus and prompt the migration of leukocytes to an injured or infected site. Such inflammatory chemokines can also activate cells to raise an immune response.

Chemokines are structurally related, because most of them contain four invariant cysteine residues. Depending on the arrangement of the first two of these cysteines, chemokines are divided into four subfamilies: CXC (alpha), CC (beta), C (gamma), and CX3C (delta) [3]. Chemokines are produced as propeptides and are cleaved during secretion to produce an active mature protein [4] that functions by activating G-protein-coupled receptors. The receptors for these chemokines have been termed accordingly as CXCR, CCR, CR, and CX3CR [5].

## 4. Hepatitis C Virus (HCV) and Immune Response

HCV is a hepatotropic, noncytopathic virus of the family Flaviviridae, which induces both acute and chronic necro-inflammatory liver disease [6, 7]. HCV escapes immune control in 60–85% of cases. When infecting the liver parenchyma, HCV continuously releases viral particles into the blood stream. The first line of defense that HCV will encounter includes natural killer (NK) cells and natural killer T (NKT) cells [8]. These cells are activated by type 1 IFN (alpha and beta) released by infected liver cells. NK and NKT cells constitute a relevant source of IFN-gamma and TNF-alpha [9]. These cytokines inhibit viral replication without destroying liver cells. NK cells are activated by IL-12 released from dendritic cells (DCs) and thus become empowered to eliminate infected cells [10]. NK cells may also induce partial or total DCs maturation [11].

DCs can process viral antigens and present them to specific immune system cells via class I and class II major histocompatibility complex (MHC) molecules. DCs capture viral particles through Toll-like receptors (TLRs). Upon activation, DCs secrete several types of cytokines (IL-12, TNF-alpha, IFN-alpha, IL-10) that will regulate and polarize the response of adjacent cells [12]. Mature DCs leave the liver after viral epitope collection and head for lymph nodes, where they will activate T cells in the specific immune system [13].

Cytokines released in the liver parenchyma induce chemokine release by liver cells, including IFN-gamma-inducible protein (IP-10/CXCL10), IFN-gamma-induced monokine (MIG/CXCL9), IFN-inducible T-cell alpha chemoattractant (I-TAC/CXCL11), macrophage inflammatory protein (MIP)-1alpha (MIP/CCL3), and MIP-1beta/CCL4, which recruit [14] specific cells capable of infection control.

Mature DCs and immature T cells, both of which express chemokine receptor CCR7, are recruited towards lymph nodes by secondary lymphoid-tissue cytokine (SLC/CCL21) [13]. In the lymph node, T cells expressing T-cell receptors (TCRs) appropriate for the recognition of epitopes presented by DCs in their MHC molecules are activated. The interaction between the TCR and MHC-viral epitope complex results in specific T-cell activation. Certain specific CD8 T cells, cytotoxic T lymphocytes (CTLs), become cytolytic, secrete type 1 cytokines, and travel to the infected liver [15–17]. Specific CD4+ T cells will regulate the adaptive response by secreting Th1 cytokines (IL-2, IFN-gamma, TNF-alpha) to facilitate a cell-mediated immune response and Th2 cytokines (IL-4, IL-10, IL-13) to regulate the humoral immunity [18]. It is widely accepted that adaptive immune response plays a key role in the control of HCV infection.

## 5. Cytokines and HCV Chronic Infection

HCV manages to escape immune response. To this end they interfere with various immune mechanisms including cytokine activity modulation.

### 5.1. Innate Immunity

A primary cell defense mechanism during initial infection is the synthesis of antiviral type 1 IFN-alpha/beta [19]. On binding its receptor, IFN-alpha/beta activates a number of intracellular mechanisms that can prevent viral replication and spread to other liver cells. HCV is a good inducer of IFN-alpha/beta expression [20]. However, HCV seems to be, at least in part, unresponsive to IFN-alpha/beta effects and may effectively replicate in the liver despite such gene induction. HCV can block type 1 IFN induction; this possibly results from the fact that nonstructural proteins (NS 3 and NS5A), and structural protein E2 may both potentially block the expression and transcription of IFN-alpha/beta-induced genes. HCV NS5A protein induces IL-8 expression, which is associated with IFN-alpha inhibition [21].

The outcome of a viral infection depends on the interplay between the host capacity to trigger potent antiviral responses and viral mechanisms that counteract them. Although Toll-like receptor (TLR)-3, which recognizes virally derived double-stranded (ds) RNA, transmits downstream antiviral signaling through the TIR adaptor Trif (TICAM-1), viral RNA-sensing RIG-like helicases (RLHs) use the mitochondrial-bound CARD protein Cardif (IPS-1/MAVS/VISA). The importance of these two antiviral signaling pathways is reflected by the fact that both adaptors are inhibited through specific cleavage triggered by the HCV serine protease NS3-4A [22, 23].

NK cells and NKT cells exert their antiviral action through direct, non-MHC-restricted cytotoxic mechanisms and IFN-gamma production [24]. In addition, they allow maturation for DCs favoring the development of Th1/Tcytotoxic (Tc)1 responses [10]. However, they do not seem to play a significant role in acute HCV infection [25]. It has been suggested that HCV can block NK cells and NKT cells functions thus preventing antiviral cytokines such as IFN-gamma from being produced, via an interaction between HCV E2 protein and NK-cell CD81 molecule [26].

During chronic infection with HCV, a decrease in IFN-alpha production by plasmacytoid DCs has been reported [27], such as a decrease in IL-12 production by myeloid DCs [28]. In fact, HCV structural proteins can interact with TLR-2 in monocytes and induce IL-10 production, which inhibits IL-12 and IFN-alpha production in DCs [29]. However, other studies reported an increased IFN-alpha production, especially in patients who fail to respond to exogenous IFN-alpha, in whom IFN-stimulated genes (ISGs) are highly activated [30].

It has been also suggested that DCs cytokine profile cannot polarize T-cell responses towards a Th1/Tc1 response [31] and contributes to inadequate NK cells and NKT cells activation. However, other studies have shown that a progressive liver injury in chronic hepatitis C infection correlates with increased intrahepatic expression of Th1-associated cytokines [32, 33].

### 5.2. Adaptive Response

HCV CD4+-T cells play a key role in adaptive response in that they provide help in activating cytotoxic and humoral responses. They can secrete Th1-cytokines including IFN-gamma, which favors neutrophil and macrophage recruitment and leads to inflammatory response. They also may release Th2 cytokines such as IL-4 and IL-10, which limit Th1 cytokine-mediated response and favor the development of humoral response [34]. A multispecific, strong, sustained, CD4+-T-cell-specific Th1 response may be seen in infections with HCV infection evolving to resolution [35]. However, when infection becomes chronic, a weak CD4-T-specific response with few specificities and scarce type 1 cytokine production is observed [36].

CD8+ CTLs can clear viruses using apoptosis-related cytolytic mechanisms and mechanisms mediated by type 1 cytokines (IFN-gamma, TNF-alpha). In chronic infection with hepatitis B virus or HCV, specific CTLs are few and engage few specific targets; they also display anergic characteristics with reduced type 1 cytokine secretion [37]. Another potential mechanism of blocked type 1 cytokine production results from regulatory T-cell activity. These cells can release IL-10 and TGF-beta and inhibit proliferation and cytokine synthesis in T cells, either directly or through other cytokines, in hepatitis C [38].

Cytokines produced by T cells play a role in the regulation of humoral responses; nevertheless, these responses cannot control chronic viral hepatitis, even though they play a role in the pathogenesis of extrahepatic manifestations [18].

## 6. Cytokines and Liver Damage

When specific immune response fails to control viral replication, the infected liver cells secrete IFN-gamma-induced chemokines such as CXC chemokine ligand CXCL9, CXCL10, and CXCL11, which result in the migration of nonspecific mononuclear cells into the liver [39], which are unable to control infection but result in sustained liver damage [40]. Inhibition of these chemokines limits nonspecific cell migration and hence reduces the inflammation [41]. The recruitment of persistent mononuclear infiltrates leads to the development of chronic inflammation, which results in sustained liver damage. Finally, chronic inflammation induces regenerating mechanisms in the liver parenchyma. Several factors influence this process, including cytokines such as IL-6, TNF-alpha, TGF-beta, hepatocyte growth factor, and epidermal growth factor. These and other factors activate transcription factors such as nuclear factor-*κ*B, signal transducer, and activator of transcription 3 which initiate the gene expression cascade leading to hepatocyte proliferation [42].

Persistent inflammation also activates hepatic stellate cells, myofibroblasts, and fibroblasts, which favors the development of liver fibrosis. The activation of these cells is regulated by pro-inflammatory cytokines such as TGF-beta, IL-6, TNF-alpha, CCL21, and platelet-derived growth factor, among other stimuli [43].

## 7. HCV-Related Extrahepatic Diseases (HCV-EHDs)

HCV is known to be responsible for both hepatic and HCV-EHDs. The most important systemic HCV-EHDs are HCV-related mixed cryoglobulinemia (MC) (MC+HCV) and lymphoproliferative disorders, while the most frequent and clinically important endocrine HCV-EHDs are autoimmune thyroid disorders (AITDs).

## 8. Cytokines, Cryoglobulinemia, and Lymphoproliferation

MC is a distinct syndrome clinically characterized by purpura, weakness, arthralgia, and involvement of one or more organ systems, including membranoproliferative glomerulonephritis, peripheral neuropathy, skin ulcers, liver damage, and diffuse vasculitis. Cryoprecipitable immunocomplexes, namelymixed (IgG-IgM) cryoglobulins, are the serological hallmark of the disease: IgG is the autoantigen and IgM, with rheumatoid factor (RF) activity, the autoantibody. MC is classified in type 2 and type 3 according to the presence of polyclonal or oligo-monoclonal IgMs. Because expansion of RF-producing B cells is the underlying disorder of MC, this condition is considered a “benign” B-cell lymphoproliferative disease [44, 45].

The mechanism(s) responsible for the lymphoproliferation surrounding MC remain unknown. Due to geographical heterogeneity in prevalence of MC+HCV, it is conceivable that unknown genetic and/or environmental factors may influence the development of this syndrome [46]. Several data are consistent with the possibility that chronic stimulation of B cells by viral epitopes could play an important role [47–49].

A wide body of evidence, in addition, strongly suggests that a key factor in the pathogenesis of MC+HCV is represented by the inhibition of the apoptosis of B cells, leading to their progressive accumulation. First, this is suggested by the histopathological characteristics of liver and/or bone marrow lymphocyte infiltrates in MC patients [50], as well as by the high prevalence of *bcl-2* rearrangement (t(14; 18) translocation) in patients with MC, with regression of translocated B-cell clones after successful antiviral therapy [45, 51, 52].

Furthermore, B-lymphocyte stimulator (BLyS) serum levels are significantly correlated with B-cell proliferation during chronic HCV infection. These results strongly suggest a role for BLyS in the induction and expression of HCV- B-cell proliferation [53–55]. Chemokine CXCL13, also known as BCA-1 (B cell-attracting chemokine-1) or BLC (B-lymphocyte chemoattractant), is a major regulator of B-cell trafficking. HCV infection may be associated with B-cell dysfunction and lymphoproliferative disorders, including MC+HCV. The results by Sansonno et al. [56] indicate that upregulation of CXCL13 gene expression is a distinctive feature of HCV-infected patients. Higher levels of this chemokine in the liver as well as in the skin of patients with active MC+HCV vasculitis suggest a possible interrelation between these biologic compartments.

Recently, Saadoun et al. [57] studied the local immune response in the liver, which is considered the principal site for immune reactions involved in MC pathogenesis. In that study, the cytokine profile of liver-infiltrating T lymphocytes from MC+HCV patients and without MC (of type 2) were compared. They showed that, although no differences were found in the proportion of CD4+, CD8+ liver T cells, the ability of freshly isolated liver T cells to produce type 1 cytokines in response to stimulation with phorbol myristate acetate and ionomycin for 6 hours was significantly higher in MC+HCV patients than in HCV-infected controls without MC, whereas production of type 2 cytokines by these cells was similar (IL-4) or reduced (IL-10).

This agrees with previous data obtained in peripheral blood mononuclear cells [58], ruling out the possibility of a discrepancy between the response of peripheral and liver T cells. Interestingly, in both studies by Saadoun et al. and Loffreda et al. [57, 58], a reduced expression of IL-10 (a strong inhibitor of IFN-gamma production) is demonstrated regardless of the different sources. These observations suggest that the evolution of HCV infection toward MC is characterized by a strong Th1 response.

Several studies have shown an increased expression of IFN-gamma [59] and IFN-gamma-inducible chemokines [60], in particular CXCL10, in hepatocytes and in lymphocytes of HCV-infected patients [61, 62], directly related with the degree of inflammation and with an increase of circulating levels of IFN-gamma and CXCL10 [14, 63–66].

Furthermore, it has been shown that NS5A and core proteins, alone or by the synergistic effect of cytokines, such as IFN-gamma and TNF-alpha, are capable of upregulating *CXCL10* and *CXCL9* gene expression and secretion in cultured human hepatocyte-derived cells [67], suggesting that CXCL10 produced by HCV-infected hepatocytes could play a key role regulating T-cell trafficking into a Th1-type inflammatory site as the liver tissue during chronic HCV infection, by recruiting Th1 lymphocytes that secrete IFN-gamma and TNF-alpha, which induce CXCL10 secretion by hepatocytes, thus perpetuating the immune cascade [68].

Furthermore, we have recently shown that circulating CXCL10, CXCL11, IFN-gamma-inducible (Th1) chemokines are higher in patients with MC+HCV than in chronic hepatitis C (CHC) patients. Moreover, our studies demonstrate markedly high serum levels of CXCL10 and CXCL11 in patients with MC+HCV compared to healthy controls in particular in the presence of active vasculitis. A strong relationship between circulating IFN-gamma and CXCL11 was shown, strongly supporting the role of a Th1 immune response in the pathogenesis of MC+HCV patients [69–74].

For comparison the prototype Th2 chemokine (C-C motif) ligand 2 (CCL2) was not significantly different in patients with MC+HCV and active vasculitis than in MC patients, and it suggests that the Th1 CXCL10 chemokine is specifically involved in the appearance of vasculitis in these patients [74].

The pro-inflammatory cytokines IL-1beta, IL-6, and TNF-alpha have also been evaluated in MC+HCV patients. In fact, MC+HCV patients show significantly higher mean IL-1beta, IL-6, and TNF-alpha levels than the controls or the HCV patients. If the importance of IL-1beta and IL-6 in the pathogenesis of MC is confirmed, these results will open the way for the evaluation of new therapies for MC [75].

On the whole the above-mentioned data underline the importance of the activation of the Th1 immunity in the immunopathogenesis of MC+HCV, but suggest a complex dysfunction of the cytokine/chemokine network in these patients, involving also pro-inflammatory cytokines.

## 9. Cytokines and AITDs Associated with HCV and MC

The pattern of thyroid disorders observed in HCV infection is characterized by the presence of increased circulating levels of antithyroid peroxidase antibody (AbTPO) and increased risk of hypothyroidism in AbTPO positive subjects [76–80].

This pattern is similar to that observed in IFN-alpha-treated patients, too [81].

Differences in geographical distribution [82], genetic variability in the populations studied [83], and environmental cofactors, such as iodine intake or other infectious agents [84, 85], could play an important role in the development of AITD.

Recently it has been shown that high levels of CXCL10 are present in patients with autoimmune thyroiditis (AT), in particular in the presence of hypothyroidism [68], and an involvement of Th1 immune response in the induction of AT [86], Graves' disease, and Graves' ophthalmopathy [87] has been shown. Furthermore, the presence of HCV in the thyroid of chronically infected patients has been recently demonstrated [88, 89]; however, other studies are needed to furtherly confirm this point.

On the above-mentioned bases, it has been speculated that HCV thyroid infection may act by upregulating CXCL10 gene expression and secretion in thyrocytes (as previously shown in human hepatocytes [67]) recruiting Th1 lymphocytes that secrete IFN-gamma and TNF-alpha, which induce CXCL10 secretion by thyrocytes, thus perpetuating the immune cascade, that may lead to the appearance of AITDs in genetically predisposed subjects.

This hypothesis has been recently confirmed by two studies that demonstrated high serum levels of CXCL10 in MC+HCV patients and showed that CXCL10 is significantly higher in the presence of AT compared to MC+HCV patients without thyroiditis [90, 91]. For comparison the prototype Th2 chemokine CCL2 was not significantly different in patients with MC+HCV in the presence of AT than in MC+HCV patients, and it suggests that the Th1 CXCL10 chemokine is specifically involved in the appearance of AT in these patients [91].

Among the pro-inflammatory cytokines, IL-1beta and TNF-alpha were not associated with the presence of AT in MC+HCV patients, while IL-6 was modestly but significantly increased in patients with AT [71, 92].

On the whole the above-mentioned data underline the importance of the activation of the Th1 immunity in the immunopathogenesis of AT in patients with MC+HCV.

## 10. Cytokines and Type 2 Diabetes Mellitus (T2DM) Associated with HCV and MC

Several clinical epidemiological studies since 1994 have reported that HCV infection is linked to diabetes [93]. The association between HCV infection, in patients without cirrhosis (a well-known risk factor for T2DM), and T2DM has been first studied in two of our studies, in patients with chronic HCV infection (HCV+) associated with MC (MC+HCV) [94] and in patients with HCV-related chronic liver disease [95].

There is one population study (National Health and Nutrition Examination Survey-NHANES III 1988–1994) that showed an adjusted odds ratio of 3.8 for T2DM for those who were aged >40 years and HCV+ [96] and increased incidence of T2DM [97].

There have been a few reports, too, that IFN treatment of HCV infection improves glucose tolerance [94, 98] when HCV infection is eradicated; however, another study did not confirm these results [99].

Altogether the above-mentioned data indicate that HCV chronic infection is a risk factor for developing T2DM.

### 10.1. Mechanism

#### 10.1.1. Insulin Resistance and Steatosis

It is speculated that insulin resistance (as a consequence of hepatic steatosis (i.e., present in about 50% of the subjects with HCV infection) [93] and/or elevated expression of TNF-alpha (strongly correlated with the degree of liver diseases and the level of insulin resistance) [89]) may lead to the development of T2DM [93].

#### 10.1.2. Direct Islet Cell Destruction by HCV

Masini et al. [100] recently demonstrated a direct cytopathic effect of HCV at the islet cell level.

#### 10.1.3. Possible Autoimmune Induction

The type of diabetes manifested by patients with HCV chronic infection is not the classical T2DM. The labelling of HCV+ patients as T2DM is purely conventional and possibly inaccurate: the lines separating type 1 diabetes from latent autoimmune diabetes in adults (LADA) and from T2DM are fading away as new pathogenetic information is obtained [101].

Three studies have previously reported [94, 95, 102] that HCV+ patients T2DM were leaner than T2DM controls and showed significantly lower LDL-cholesterol and systolic and diastolic blood pressure. Furthermore, MC-HCV+ patients with T2DM had non-organ-specific autoantibodies more frequently (34% versus 18%) than nondiabetic MC-HCV+ patients [94].

An immune-mediated mechanism for MC-HCV+ associated diabetes has been postulated [94], and a similar pathogenesis might be involved in the diabetes of HCV+ patients. This hypothesis is strengthened by the finding that autoimmune phenomena in T2DM patients are more common than previously thought [103]. Since the prevalence of classic beta-cell autoimmune markers in HCV+ patients has not been found to be increased [89], other immune phenomena might be involved [104].

On the above-mentioned bases, it could be interesting to speculate that HCV infection of beta cells [100] may act by upregulating CXCL10 gene expression and secretion (as previously shown in human hepatocytes) recruiting Th1 lymphocytes that secrete IFN-gamma and TNF-alpha, which induce CXCL10 secretion by beta cells, thus perpetuating the immune cascade that may lead to the appearance of beta cells dysfunction in genetically predisposed subjects.

This hypothesis has recently been confirmed by a study that demonstrates higher serum levels of CXCL10 in HCV+ patients with T2DM with respect to those without [64, 105].

## 11. Therapeutic Role of Cytokines in Chronic Viral Hepatitis

IFN-alpha is the only cytokine currently used in the treatment of chronic viral hepatitis. In CHC, pegylated IFN-alpha combined with ribavirin leads to sustained viral clearance in 50% of patients [106]. The most important effect of IFN-alpha is directly antiviral; however, it has also immunomodulating actions that favor Th1/Tc1 response restoration [107–109]. On the other hand, ribavirin, a wide-spectrum antiviral agent used in combination therapy for hepatitis C, has immunomodulating effects that induce type 1 cytokine production [110]. Sustained viral load reduction with antiviral agents has also been seen to facilitate specific T response recovery with type 1 cytokine production in hepatitis C [111].

An exogenous administration of Th1-inducing cytokines such as IL-12 [112] or anti-inflammatory cytokines such as IL-10 has also been attempted to reduce intrahepatic inflammation severity [113]. However, such therapies remain experimental, and their effectiveness is unclear.

From a theoretical standpoint Tc1-associated chemokine receptors may represent an interesting therapeutic target in the development of drugs for patients with chronic hepatitis unresponsive to antiviral agents, their aim being a reduction of liver inflammation and progression to fibrosis by blocking inflammatory cell migration into the liver [39, 41].

Treatment-induced and spontaneous clearance of HCV infection are affected by various host factors. Polymorphisms in the region of the gene *IL-28B* are associated with HCV clearance, implicating the gene product, IFN-lambda3, in the immune response to HCV. Although it is not clear how the IL-28B haplotype affects HCV clearance, IFN-lambda3 upregulates IFN-stimulated genes, similar to IFN-alpha and -beta, but via a different receptor. There is also evidence that IFN-lambda3 affects the adaptive immune response.

It is known that IL-28B may establish a robust T-cell adaptive immune response [114, 115]. This effect may explain the relationship between single-nucleotide polymorphism (SNPs) near IL-28B, adaptive response, and viral clearance [116].

The IL-28B genotype can be considered, along with other factors, in predicting patient responses to therapy with pegylated IFN-alpha and ribavirin [117, 118].

Clinical studies assessing safety and efficacy in the treatment of HCV with exogenous IFN-lambda3 are currently underway. Early results suggest that IFN-lambda3 treatment inhibits HCV replication and is associated with a limited side effect profile. However, hepatotoxicity in both healthy volunteers and HCV-infected patients has been described [119].

## 12. Conclusion

Cytokines are intercellular mediators involved in viral control and liver damage as induced by infection with HCV. The complex cytokine network operating during initial infection allows a coordinated, effective development of both innate and adaptive immune responses. However, HCV interferes with cytokines at various levels and escape immune response by inducing a Th2/Tc2 cytokine profile. Inability to control infection leads to the recruitment of inflammatory infiltrates into the liver parenchyma by IFN-gamma-inducible CXCL9, -10 and -11 chemokines, which results in sustained liver damage and eventually in liver cirrhosis; however, fibrogenesis may also follow distinct paths. The most important systemic HCV-EHDs—MC, lymphoproliferative disorders, and AITDs—are associated with a complex dysregulation of the cytokine/chemokine network, involving pro-inflammatory and Th1 chemokines. The therapeutical administration of cytokines such as IFN-alpha may result in viral clearance during persistent infection and reverts this process.
